# Building an optics and photonics research ecosystem in South Korea: Collaborative innovation between academia and industry

**DOI:** 10.1038/s41377-023-01332-x

**Published:** 2023-12-04

**Authors:** Younghwan Yang, Trevon Badloe, Duheon Song, Seongjin Park, Junsuk Rho

**Affiliations:** 1 Department of Mechanical Engineering, Pohang University of Science and Technology (POSTECH), Pohang, 37673 Republic of Korea; 2 Graduate School of Artificial Intelligence, Pohang University of Science and Technology (POSTECH), Pohang, 37673 Republic of Korea; 3 Samsung Advanced Institute of Technology (SAIT), Suwon, 16678 Republic of Korea; 4 Pohang Iron and Steel Company (POSCO), Pohang, 37859 Republic of Korea; 5 Department of Chemical Engineering, Pohang University of Science and Technology (POSTECH), Pohang, 37673 Republic of Korea; 6 POSCO-POSTECH-RIST Convergence Research Center for Flat Optics and Metaphotonics, Pohang, 37673 Republic of Korea; 7 Present Address: School of System Semiconductor Engineering, Yonsei University, Seoul, 03722 Republic of Korea

**Keywords:** Nanophotonics and plasmonics, Metamaterials

**Fig. 1:**
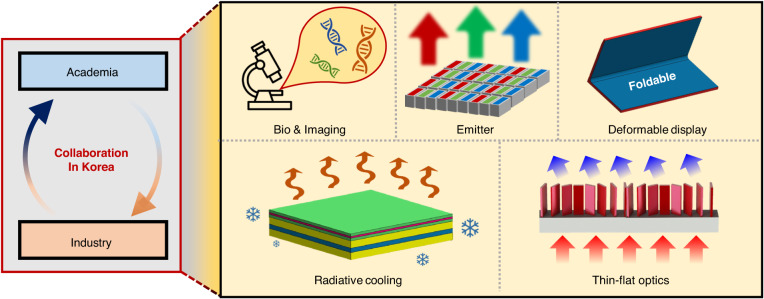
Collaboration in Korea academia and industry. Collaborative innovation has been carried out in diverse fields including biophotonics, imaging, emitters, deformable displays, radiative cooling, and thin-flat optics within the Korean industry and research community

Photonics research is a rapidly growing field that has found applications in various aspects of our lives, ranging from communication to medical imaging^1–5^. Collaborative innovation between academia and industry has been a driving force behind the significant advances in photonics research in South Korea. In this special issue, we present cutting-edge research in photonics, highlighting the importance of collaboration between academia and industry (Fig. 1). This issue covers a wide range of topics in photonics including biomedical applications^6–8^, imaging^9–11^, micro light emitting diodes (μLED)^12^, quantum dot emitters^13^, deformable displays^14^, thin-flat optics^15,16^, radiative cooling^17^, and 2D optical devices^18–20^. Additionally, we introduce upcoming disruptive photonic applications with periodically arranged structures and their integration with conventional optics^15,21^. By uniting researchers from academia and industry, we hope to foster a culture of collaboration and innovation in photonics research in South Korea, paving the way for practical applications in various fields. This special issue can serve as a reference for the current and future research directions of photonics in South Korea.

Recent advancements in biophotonics have led to the development of novel materials and techniques for various biomedical applications. One such material is a upconversion materials, which have shown promise in photochemical tissue bonding^6^. In addition, a computational strategy based on deep neural networks has been developed to reconstruct high-density super-resolution images from a few raw images, thereby improving temporal and spatial resolution in various biological and medical modalities^7^. Furthermore, microscopy-based frameworks that identify pathogens from single to few cells have been proposed as a potential solution to the long turnaround time of routine microbial identifications^8^. These innovative approaches hold great potential for advancing biophotonics and improving medical technologies.

Similarly, Korean academia has made continuous advancements in scanning technology to address current limitations and challenges. These developments aim to capture fast and intricate mechanical dynamics in real-time, enabling direct time-domain imaging of displacements and mechanical motions. For example, a line-scan time-of-flight (TOF) camera has been developed using electro-optic sampling with a frequency comb, which simultaneously measures TOF changes of more than 1,000 spatial coordinates with sub-nanometer axial resolution and a field-of-view of over several millimeters^10^. This unique combination of performance enhancements enables fast and precise imaging of both complex structures and dynamics in 3D devices and mechanical resonators. Additionally, a novel reference-free 3D polarization-sensitive computational imaging technique, termed polarization-sensitive intensity diffraction tomography, has been developed^11^. It enables the reconstruction of the 3D anisotropy distribution of both weakly and multiple scattering specimens from multiple intensity-only measurements. These advancements in scanning technology open up new possibilities for investigating the nature of anisotropic materials and studying the full-field structural and dynamic behaviors of micro- and nano-scale devices.

In the field of sensing, a novel sensing strategy using a single μLED-embedded photoactivated gas sensor has been proposed^12^. By utilizing time-variant illumination equipped with a deep neural network, the system is able to selectively identify and quantify the concentrations of various target gases with high accuracy. This approach may significantly improve the efficiency of electronic sensing technology in terms of cost, space, and power consumption. Meanwhile, a paradigm-shifting photonic crystal (PhC) phosphor platform has been further improved by increasing the refractive index contrast and planarizing the surface^13^. The upgraded PhC phosphor exhibits unprecedentedly high absorption and emission efficiency, which may further spur the development of structural engineering of phosphor materials.

Furthermore, collaborative innovation between Korean academia and industry has led to significant advancements in practical applications, including metasurface-integrated optical components^15^, large-area metaholograms^16^, hollow cavities^21^, and deformable displays^14^. Metasurfaces offer versatile capabilities for controlling electromagnetic waves and ongoing research aims to integrate them with standard optical components for commercialization^15^. One-step nanoprinting with ZrO_2_ particle-embedded resin enalbes scalable production of metaholograms in the ultraviolet^16^. Hollow cavities have unique optical characteristics and can be tailored to specific spectra and applications, making them promising platforms for optoelectronic devices^21^. Deformable displays have already been commercialized, and efforts are being made to develop 3D free-form displays for use in tactile sensation, artificial skin, and on-skin or implantable displays^14^. Industrial commercialization of these technologies presents several challenges and prospects.

Research in Korean industries is increasingly being geared towards environmental considerations. One notable area of research is radiative cooling^17^, a passive energy-free cooling technology. The review on radiative cooling in this issue explores the fundamentals of thermodynamic heat transfer that motivate radiative cooling, and discusses various photonic structures inspired by nature and their associated design procedures. The integration of photonic structures with new functionalities has also enhanced the efficiency of radiative cooling technologies, enabling applications such as reducing cooling loads in vehicles, increasing the power generation of solar cells, and personal thermal regulation. The article concludes with a discussion on emerging issues and potential solutions for the future of radiative cooling.

Moreover, Korean researchers have focused on the application of 2D Dirac materials in optoelectronics, harnessing the collective oscillations of massless particles to create atomically thin devices with exceptional optoelectronic functions^18^. Graphene and 3D topological insulators have shown promising results in terms of mid-infrared and terahertz radiation confinement, enabling advancements in bio-molecular sensors, photodetectors, and laser-driven light sources. Additionally, an electrical and spectral method of resolving chiral exceptional points in a non-Hermitian gated graphene metasurface has been proposed, leading to the observation of enhanced asymmetric polarization conversion^19^. The authors anticipate that electrically controllable non-Hermitian metasurface platforms can serve as an interesting framework for the investigation of rich non-Hermitian polarization dynamics around chiral exceptional points.

Another area of interest in Korea is the design and manufacturing of ultra-thin graphene optics, such as planar diffractive lenses (PDL) that can be patterned on ultra-thin and flexible substrates and conformally attached to arbitrarily shaped surfaces, making them ideal for compact and lightweight optical systems for various applications^20^. The use of direct laser writing of laser-induced-graphene is actively being applied to the patterning of PDLs, offering high design flexibility, low process complexity, and a chemical-free process with reasonable investment cost. In-depth studies on photon-material interactions with different laser parameters have enabled the production of exemplary laser-written 1D and 2D PDL structures using various base materials. The combination of ultra-thin PDLs with conventional bulk refractive or reflective optical elements has the potential to leverage the advantages of each optical element and enable the realization of hybrid PDLs for various industries, including micro-electronics surface inspection, biomedical applications, outer space exploration, and extended reality industries.

In the field of ophthalmology, the development of multifocal and extended depth of focus intraocular lenses (IOLs) has revolutionized vision correction^9^. These IOLs, embedding micro-thin geometric phase (GP) lens layers, surpassing conventional diffractive IOLs in terms of multifocality while avoiding the need for additional surface patterns. By adjusting the number of stacked GP layers and their thickness, the GP IOLs can achieve various focal points and light splitting ratios, providing enhanced vision capabilities.

As mentioned earlier, South Korea has fostered extensive industry-academia collaboration research, with a particularly focus on the next-generation display and nanophotonic devices. One notable example is the POSCO-POSTECH-RIST convergence research center for flat optics and metaphotonics, which plays a pivotal role in bridging the gap between science and technology by promoting the academia-industry collaboration model in South Korea. The center has already proposed massive production process of large-area metalenses at the visible spectrum^22^ and organized integrated metasurfaces for commercialization^15^. In addition to POSCO, other domestic companies in South Korea are increasingly demonstrating interest in academia-industry collaboration and startup initiatives, embracing open innovation. Through such collaborations, these companies are seeking technological innovation in their existing businesses and exploring new ventures. This growing trend in South Korea signifies the importance of fostering partnerships and promoting open innovation to drive both technological advancements and the discovery of new business opportunities.

In conclusion, the research presented in this special issue highlights the crucial role of collaboration between academia and industry in driving the advancements of photonics research. The innovative approaches showcased here have significant potential for advancing biophotonics, exploring the characteristics of anisotropic materials, and investigating the structural and dynamic behaviors of micro- and nano-scale devices. Furthermore, the practical industry-based research presented in this issue, such as metasurface-integrated optical components^15^, hollow cavities^21^, and deformable displays^14^, holds great promise for commercialization. We are optimistic that this special issue will inspire further collaboration and innovation in photonics research in South Korea, ultimately leading to more real-life applications and technological advancements in various fields. Additionally, the emphasis on environmental sustainability, as exemplified by research on radiative cooling^17^, demonstrates a commitment to environmentally-friendly and energy-efficient technologies.
